# Methylglyoxal Mediates the Association Between 2-Hour Plasma Glucose and HbA1c With Inflammation: The Maastricht Study

**DOI:** 10.1210/clinem/dgae640

**Published:** 2024-09-24

**Authors:** Dijia Sun, Marleen M J van Greevenbroek, Jean L J M Scheijen, Jaycey Kelly, Casper G Schalkwijk, Kristiaan Wouters

**Affiliations:** Department of Internal Medicine, Maastricht University Medical Center, Maastricht, The Netherlands; CARIM School for Cardiovascular Diseases, Maastricht, The Netherlands; Department of Internal Medicine, Maastricht University Medical Center, Maastricht, The Netherlands; CARIM School for Cardiovascular Diseases, Maastricht, The Netherlands; Department of Internal Medicine, Maastricht University Medical Center, Maastricht, The Netherlands; CARIM School for Cardiovascular Diseases, Maastricht, The Netherlands; Department of Internal Medicine, Maastricht University Medical Center, Maastricht, The Netherlands; CARIM School for Cardiovascular Diseases, Maastricht, The Netherlands; Department of Internal Medicine, Maastricht University Medical Center, Maastricht, The Netherlands; CARIM School for Cardiovascular Diseases, Maastricht, The Netherlands; Department of Internal Medicine, Maastricht University Medical Center, Maastricht, The Netherlands; CARIM School for Cardiovascular Diseases, Maastricht, The Netherlands

**Keywords:** diabetes, methylglyoxal, cardiovascular complications, inflammation

## Abstract

**Context:**

Glucose excursions in persons with diabetes may drive chronic inflammation. Methylglyoxal (MGO) is formed from glucose, is elevated in persons with diabetes, and is a potent glycating agent linked with inflammation.

**Objective:**

We investigated whether glucose excursions are associated with low-grade inflammation and whether MGO mediates this association.

**Methods:**

We used data from The Maastricht Study, an extensive phenotyping study into the etiology of type 2 diabetes and its complications. Data of 3017 participants, who underwent an oral glucose tolerance test and where data on MGO levels and inflammation were available, were used. Linear regression analyses, adjusted for potential confounders, evaluated associations between fasting plasma glucose (FPG), 2-hour plasma glucose (2h-PG) and HbA1c, and low-grade inflammation (stdβ, [95% CI]) were calculated from plasma concentrations of C-reactive protein, serum amyloid A, interleukin-6, interleukin-8, tumor necrosis factor, and soluble intercellular adhesion molecule-1. Mediation analyses investigated whether MGO mediated these associations.

**Results:**

2h-PG (0.172, [0.110; 0.234]) and HbA1c (0.148, [0.101; 0.196]), but not FPG (0.049, [−0.002; 0.100]), were associated with low-grade inflammation. 2h-PG and HbA1c were also associated with 2h-MGO (0.471, [0.407; 0.534], and 0.244, [0.195; 0.294], respectively). Furthermore, 2h-MGO was independently and positively associated with low-grade inflammation (0.078, [0.037; 0.120]). 2h-MGO mediated 23% of the association between 2h-PG and inflammation, and 16% of the association between HbA1c and inflammation.

**Conclusion:**

MGO mediates the association between postload glucose excursions and HbA1c with inflammation, providing evidence for a role of postprandial MGO formation to hyperglycemia-induced low-grade inflammation.

The prevalence of diabetes is increasing yearly, aggravating human microvascular and macrovascular disease, and causing death worldwide (1). Hyperglycemia-induced inflammation is considered an important contributor to the severe vascular complications of diabetes (2). Postprandial glucose excursions in persons with diabetes are proposed to be more important determinants of cardiovascular disease (CVD) than fasting plasma glucose levels (FPG) (2, 3). Our experimental data in mice showed that glucose peaks induce inflammation and concomitant atherosclerosis (4). In humans, both high glucose peaks and high 2-hour plasma glucose (2h-PG) levels were associated with the highest CVD risk compared with the individuals with the lowest plasma glucose peaks (5). Glycosylated hemoglobin A1c (HbA1c), which reflects overall blood glucose control including high glucose excursions in the previous days and weeks, is more strongly associated with risk of CVD and all-cause mortality than fasting plasma glucose (FPG) (6), although HbA1c has limitations for correct interpretation of glycemic control because of interindividual differences (eg, in erythrocyte turnover and glucose gradient across erythrocyte membranes) (7). Moreover, postprandial glucose fluctuations were shown to induce endothelial dysfunction (8, 9) and an acute increase in plasma glucose induces elevated inflammatory cytokine concentrations in humans (10).

A potential intermediate step in the association between postprandial glucose fluctuations and vascular disease is the formation of methylglyoxal (MGO) (a highly reactive dicarbonyl and a major precursor in the formation of advanced glycation endproducts [AGEs]) (11). MGO can affect inflammation, either directly by inducing reactive oxygen species and activation of NF-κB inflammatory signaling, or indirectly via AGE formation and subsequent binding to the receptor for AGEs (RAGEs) (12, 13). The main source of endogenous MGO is its glucose-dependent formation as a byproduct of glycolysis. We previously showed that plasma MGO is associated with low-grade inflammation and that higher plasma concentrations of MGO are prospectively associated with albuminuria, estimated glomerular filtration rate, and retinopathy, as well as with incident CVD (14). Furthermore, we have shown that MGO concentrations increase after an oral glucose tolerance test (OGTT) and after a mixed meal test, and that these MGO excursions are more pronounced in persons with a disturbed glucose metabolism (15, 16). In addition, we have shown that MGO formation after an OGTT is completely derived from exogenous glucose (17). Most importantly, we found that repeated MGO peaks induce the production of myeloid cells and worsens atherosclerosis in mice (18). Collectively, these data suggest a role for glucose-induced MGO formation in the development of hyperglycemia-induced inflammation. We therefore hypothesized that postload glucose excursions induce plasma MGO, which, in turn, provokes chronic low-grade inflammation. We investigated these hypotheses in the Maastricht Study, a large, population-based observational human cohort, enriched for type 2 diabetes mellitus (T2DM).

## Materials and Methods

### Study Design and Participants

We used data from the Maastricht study, an observational prospective population-based cohort study that was initiated in 2010 to investigate the development and progression of T2DM and associated factors with an oversampling of individuals with T2DM (19). A flowchart of the inclusion of study participants in the current analyses is presented elsewhere (Figure S1 (20)). The present report includes cross-sectional data from the first 7689 participants, living in the southern part of The Netherlands aged 40 to 75 years, who completed the baseline survey between November 2010 and December 2017. Measurements were performed by trained researchers according to standardized protocols during 3 visits of 3 to 4 hours length at the Maastricht Research Center. All participants’ measurements were taken during these 3 visits within a 3-month timeframe. Outcome variables and covariates of interest were measured via questionnaires, interviews, physical examinations, and blood sampling. The study has been approved by the institutional medical ethics committee (NL31329.068.10) and the Minister of Health, Welfare and Sports of The Netherlands (Permit 131088-105234-PG). All participants gave written informed consent.

### Main Dependent Variables: Biomarkers of Low-grade Inflammation

Inflammatory markers, namely, C-reactive protein (CRP), serum amyloid A, interleukin (IL)-6, IL-8, tumor necrosis factor (TNF), and serum intercellular adhesion molecule-1 were quantified in EDTA plasma on commercially available immunoassays (Meso Scale Discovery), as described before (14).

### Independent Variables: Fasting and Post-OGTT Glucose Concentrations and HbA1c

All participants underwent a standardized 75-g OGTT after overnight fasting, except for those who used insulin or had a fasting value greater than 11.0 mmol/L (19). Fasting (t = 0 minutes) and 2-hour glucose (t = 120 minutes) concentrations were used to classify participants into 3 categories according to the criteria of the WHO 2006: normal glucose metabolism (NGM), prediabetes (impaired fasting glucose and/or impaired glucose tolerance), and T2DM. HbA1c concentrations were measured in fasting venous blood samples using standard clinical chemistry techniques (19).

### Plasma MGO Concentration

Plasma concentrations of MGO were measured at time point 0 (fasting) (F-MGO) and 120 minutes (2h-MGO) of the OGTT by ultrahigh performance liquid chromatography and tandem mass spectrometry (21).

### Evaluation of Covariates

Covariates were measured via physical examination, questionnaires, and interviews (19). Body mass index (BMI) and systolic blood pressure were measured during the physical examination. Age, sex, physical activity (CHAMPS questionnaire), educational status (low, medium, high), history of cardiovascular disease (yes, no), smoking behavior (never, former, current), and type of medication taken (antihypertensive, lipid-lowering, blood glucose–regulating medications) were assessed via questionnaires and interviews (19). A validated food frequency questionnaire (22) was administered to estimate adherence to the Dutch dietary guidelines based on the Dutch Healthy Diet (DHD) index (23).

### Statistical Analysis

Normally distributed variables are presented as mean ± SD, skewed variables as median with interquartile range, and categorical variables as proportions (%). Analysis of variance was used for group comparisons of continuous variables, and Χ^2^ test in case of categorical variables. Non-normally distributed variables were Ln-transformed prior to further analyses.

Multiple linear regression was used to investigate the relationship between FPG, 2h-PG, and HbA1c (independent variables) and a low-grade inflammation score (dependent variable). 2h-PG was used as an estimate of postprandial glucose excursion. FPG, 2h-PG, and HbA1c were Z-standardized to enable direct comparison of the effect sizes. A low-grade inflammation score was calculated by first performing Ln-transformation and Z-standardization of each of the 6 inflammatory factors, then averaging them, and lastly Z-standardizing the resulting overall score. In case of subgroup analyses, inflammation scores were calculated separately based on the number of participants in a subgroup.

Results are expressed as regression coefficients and corresponding 95% CIs. Model 1 is the crude model; model 2 is adjusted for age (in years), sex (male/female), physical activity (self-reported in hours/week), DHD index (sum score), education level (low, medium, high, as dummy variables), and smoking status (never, former, current, as dummy variables); model 3 is additionally adjusted for diabetes status (NGM, prediabetes, T2DM, as dummy variables), systolic blood pressure (mmHg), and medication use (glucose lowering, lipid-modifying, and antihypertensive medication, each yes/no). The main model, model 4, was additionally adjusted for BMI (kg/m^2^).

Mediation analysis was performed if the associations in the main analyses were significant. Herein, we investigated whether the association of FPG, 2h-PG, and/or HbA1c with the low-grade inflammation score was mediated by MGO measured in the fasting state, T = 0 minutes (F-MGO), and at T = 120 minutes (2h-MGO), respectively. Since HbA1c is primarily determined by postprandial blood glucose levels in the previous 6 to 8 weeks, 2h-MGO was used as mediator in the association between HbA1c and inflammation. The main models described above served as the c-path (ie, exposure–outcome); the associations of FPG with F-MGO, and of 2h-PG and HbA1c with 2h-MGO as a-path (ie, exposure–mediator); and F-MGO and 2h-MGO with the low-grade inflammation score as b-path (ie, mediator–outcome). For all mediation analyses, the regression-based approach was used to estimate the direct (c′-path) and indirect (a × b-path) effects. The mediation analyses were adjusted for the same confounders as in the main regression analyses shown above. The b-path was additionally adjusted for FPG, 2h-PG, or HbA1c, as appropriate.

Interaction analyses were performed for sex and for T2DM and prediabetes status by incorporating interaction terms (eg, age × sex) in the regression models. Stratified analyses were performed when interaction terms were statistically significant.

To evaluate the robustness of our findings, the main analyses were repeated while excluding participants with prevalent CVD. Moreover, we repeated the analyses using the 6 inflammatory markers in separate analyses to evaluate if the observed associations were contributable to a single inflammation marker.

Statistical analyses were performed using IBM SPSS Statistics version 27.0. *P* < .05 was considered to be statistically significant, for main affects and for interaction. Mediation analyses were performed using the SPSS plug-in tool PROCESS v4.0 Analytical Modeling Tool (24). The 95% CIs of the mediated effect were estimated using nonparametric bootstrapping (5000 samples).

## Results

### General Characteristics of the Study Population

Table 1 shows the characteristics of the population stratified according to glucose metabolism status (NGM, prediabetes, T2DM). The overall population had a mean age of 59.9 ± 8.2 years, 49.5% were women, and 58.5% had NGM, 15.0% had prediabetes, and 26.5% had T2DM. F-MGO and 2h-MGO as well as the plasma markers of low-grade inflammation were higher in participants with prediabetes and T2DM. Participants with T2DM had, by definition, higher FPG, 2h-PG, and HbA1c. Participants with prediabetes and T2DM showed a gradually lower DHD15 score and self-reported total physical activity, while systolic blood pressure and BMI were higher.

**Table 1.** General characteristics of the overall study population (n = 3017), according to diabetes status

Characteristic		NGM n = 1766	Prediabetes n = 453	Type 2 diabetes n = 798	Overall*^a^*	*P* value
Age (years)*^a^*		58.2 ± 8.1	61.7 ± 7.8	62.6 ± 7.7	59.9 ± 8.2	<.001
Sex (n, % male)*^a^*		750 (42.5)	237 (52.3)	538 (67.4)	1525	<.001
Smoking status (3 categories)*^a^*(n, % diabetes status)						
Never		718 (40.7)	136 (30.0)	227 (28.4)	1081	
Former		847 (48.0)	268 (59.2)	461 (57.8)	1576	<.001
Current		201 (11.4)	49 (10.8)	110 (13.8)	360	
Education level categories*^a^*(n, % diabetes status)						
Low		464 (26.3)	162 (35.8)	354 (44.4)	980	<.001
Medium		492 (27.9)	128 (28.3)	228 (28.6)	848	
High		810 (45.9)	163 (36.0)	216 (27.1)	1189	
Lipid-modifying medication*^a^*(n, % diabetes status)						
Yes		308 (17.4)	156 (34.4)	593 (74.3)	1057	
Glucose-lowering medication*^a^*(n, % diabetes status)						
Yes		0 (0)	0 (0)	614 (76.9)	614	<.001
Blood pressure–lowering medication*^a^*(n, % diabetes status)						
Yes		393 (22.3)	204 (45.0)	569 (71.3)	1166	<.001
Dutch Healthy Diet (score)*^a^*		85.2 ± 14.7	82.9 ± 14.9	80.0 ± 14.3	83.5 ± 14.8	<.001
BMI (kg/m^2^)*^a^*		25.5 ± 3.6	27.7 ± 4.3	29.9 ± 4.9	27.0 ± 4.5	<.001
Systolic blood pressure (mmHg)*^a^*		130.8 ± 17.2	137.3 ± 17.0	142.3 ± 17.6	134.8 ± 18.0	<.001
Self-reported total physical activity (score)*^a^*		14.8 ± 8.1	14.1 ± 8.1	12.3 ± 8.1	14.0 ± 8.2	<.001
CRP (ug/mL)*^a^*		2.1 ± 3.8	3.5 ± 7.9	3.5 ± 8.6	2.7 ± 6.1	<.001
IL-6 (pg/mL)*^a^*		.7 ± 1.8	1.0 ± 2.6	1.3 ± 4.2	.9 ± 2.7	<.001
IL-8 (pg/mL)*^a^*		4.7 ± 10.5	5.7 ± 12.8	6.1 ± 6.3	5.2 ± 10.0	<.001
TNF (pg/mL)*^a^*		2.3 ± 2.8	2.3 ± .7	2.6 ± 1.1	2.4 ± 2.2	<.001
SAA (ug/mL)*^a^*		5.0 ± 11.3	7.6 ± 19.4	8.0 ± 23.7	6.2 ± 16.8	<.001
sICAM1 (ng/mL)*^a^*		332.9 ± 78.2	360.2 ± 96.1	385.0 ± 120.6	350.8 ± 96.2	<.001
FPG (mmol/L)*^b^*		5.9 ± .4	5.9 ± .6	7.9 ± 2.1	6.0 ± 1.6	<.001
2h-PG (mmol/L)*^b^*		5.4 ± 1.1	8.2 ± 1.7	14.5 ± 3.8	7.8 ± 4.2	<.001
HbA1c (mmol/mol)*^b^*		35.9 ± 3.8	38.7 ± 4.5	51.7 ± 11.5	40.5 ± 9.6	<.001
F-MGO (nmol/L)*^b^*		321.2 ± 83.4	346.0 ± 97.1	373.0 ± 131.4	338.6 ± 102.6	<.001
2h-MGO (nmol/L)*^b^*		275.4 ± 75.7	311.9 ± 86.1	384.8 ± 101.8	305.2 ± 94.6	<.001

Data are presented as mean ± SD (continuous variables) or proportion (%, categorical variables).

Abbreviations: 2h-MGO, 2 hour plasma methylglyoxal; 2h-PG, 2 hour plasma glucose; BMI, body mass index (kg/m^2^); CRP, C-reactive protein; DHD score, Dutch Healthy Diet score; F-MGO, fasting methylglyoxal; FPG, fasting plasma glucose; HbA1c, glycosylated hemoglobin A1c; IL, interleukin; NGM, normal glucose metabolism; SAA, serum amyloid A; SBP, systolic blood pressure; sICAM1, serum intercellular adhesion molecule-1; TNF-α, tumor necrosis factor-α.

^*a*^Overall participants for analysis; n = 3017.

^*b*^FPG is available for n = 3015; 2h-PG is available for n = 2828; HbA1c is available for n = 3010; F-MGO is available for n = 2670; 2h-MGO is available for n = 2461.

### Associations of FPG, 2h-PG, and HbA1c With Low-grade Inflammation

The associations of FPG, 2h-PG, and HbA1c with low-grade inflammation are presented in Table 2. In the crude analyses, all glucose measures were positively associated with low-grade inflammation. For FPG, this association was strongly attenuated and no longer significant after adjustment for potential confounders, while for 2h-PG and HbA1c the associations remained in the fully adjusted model (βs, 95% CI were 0.172, 0.110; 0.234, and 0.14, 0.101; 0.196, respectively). 2h-PG was also positively associated with all individual markers of low-grade inflammation, while HbA1c was not significantly associated with TNF and IL-6 (Table S1 (20)).

**Table 2.** Association between FPG, 2h-PG, HbA1c, and the low-grade inflammation score

Independent	Crude model		Fully adjusted model	
	β (95% CI)	*P* value	β (95% CI)	*P* value
FPG*^a^*	0.309 (0.275; 0.343)	<.001	0.049 (−0.002; 0.100)	.060
2h-PG*^b^*	0.334 (0.299; 0.369)	<.001	0.172 (0.110; 0.234)	<.001
HbA1c*^c^*	0.341 (0.308; 0.375)	<.001	0.148 (0.101; 0.196)	<.001

Table 2 shows associations in standardized regression, expressed as linear regression coefficients (95% CIs), for the following models:

Crude Model: crude association.

Fully adjusted model: adjusted for age and sex, physical activity, Dutch Healthy Diet score, education level and smoking status, systolic blood pressure, glucose-lowering drugs, blood pressure–lowering drugs, lipid-lowering drugs, diabetes status, and body mass index.

Abbreviations: 2h-PG, 2 hour plasma glucose; FPG, fasting plasma glucose; HbA1c, glycosylated hemoglobin A1c.

^*a*^FPG, inflammation score is available for n = 3015.

^*b*^2h-PG, inflammation score is available for n = 2828.

^*c*^HbA1c, inflammation score is available for n = 3010.

Next, we evaluated if these associations differed according to sex or diabetes status. Interaction was only found for T2DM (*P*_interaction_ = .005). Stratifying for presence of T2DM showed that the association between FPG and low-grade inflammation was positive in participants with T2DM, but inverse in those who do not have T2DM (Table S2 (20)). Sensitivity analyses, in which participants with known CVD were excluded, did not materially alter the results (Table S4 (20)).

### MGO Is a Mediator in the Association of 2h-PG and HbA1c With Low-grade Inflammation

In the whole study population 2h-PG and HbA1c were significantly associated with low-grade inflammation. Therefore, mediation analyses were restricted to those associations. Since HbA1c determined by postprandial blood glucose levels in the previous 6 to 8 weeks, we used 2h-MGO levels for these analyses. To assess whether all conditions for mediation analysis were met, we first investigated the associations of 2h-PG and HbA1c with 2h-MGO (a-path) and for 2h-MGO and low-grade inflammation (b-path) (Table 3). These analyses showed that 2h-PG and HbA1c were significantly and positively associated with 2h-MGO, β=0.471, 0.407; 0.534, and β=0.244, 0.195; 0.294, respectively. Furthermore, 2h-MGO was independently and positively associated with low-grade inflammation, also after adjusting for 2h-PG (β=0.078, 0.037; 0.120, or for HbA1c (β=0.082, 0.042; 0.123). For the individual markers of low-grade inflammation, the associations remained significant for IL-8 and TNF after adjustment for 2h-PG and for IL-8, TNF, and CRP after adjustment for HbA1c (Table S3 (20)).

**Table 3.** Association of 2h-PG and HbA1c with 2h-MGO, for 2h-MGO with the low-grade inflammation score

			Crude model		Fully adjusted model	
	Independent	Dependent	β (95% CI)	*P* value	β (95% CI)	*P* value
2h-PG*^a^*						
a-path	2h-PG	2h-MGO	0.555 (0.522; 0.588)	<.001	0.471 (0.407; 0.534)	<.001
b-path*^c^*	2h-MGO	Inflammation score	0.234 (0.195; 0.272)	<.001	0.078 (0.037; 0.120)	<.001
HbA1c*^b^*						
a-path	HbA1c	2h-MGO	0.476 (0.441; 0.511)	<.001	0.244 (0.195; 0.294)	<.001
b-path*^d^*	2h-MGO	Inflammation score	0.233 (0.195; 0.272)	<.001	0.082 (0.042; 0.123)	<.001

The table shows associations in standardized regression, expressed as linear regression coefficients (95% CIs). Path a shows the magnitude of the associations between 2h-PG, HbA1c, and 2h-MGO; path b shows the magnitude of the associations between 2h-MGO and the low-grade inflammation score.

Crude model: unadjusted.

Fully adjusted model: adjusted for age, sex, physical activity, Dutch Healthy Diet score, education, smoking, systolic blood pressure, glucose-lowering, blood pressure lowering–, and lipid-modifying medication, diabetes status and body mass index (kg/m^2^).

Abbreviations: 2h-MGO, 2 hour plasma methylglyoxal; 2h-PG, 2-hour plasma glucose; HbA1c, glycosylated hemoglobin A1c.

^*a*^2h-PG: n = 2461.

^*b*^HbA1c: n = 2455.

^*c*^Additionally adjusted for 2h-PG.

^*d*^Additionally adjusted for HbA1c.

**Figure 1.**
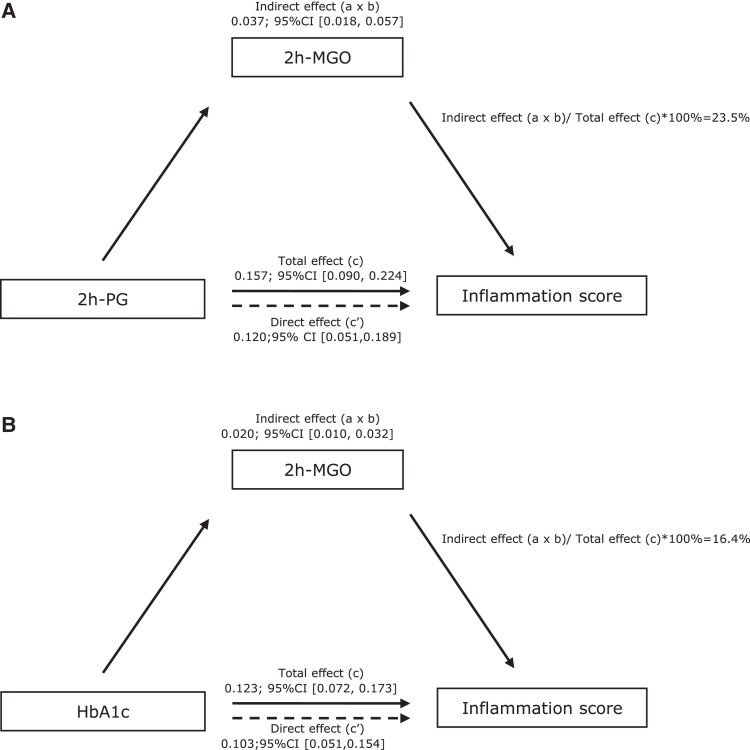
Mediation analyses: 2h-MGO as a mediator in the associations of 2h-PG and HbA1c with the low-grade inflammation score. Fully adjusted associations between 2h-PG (A) or HbA1c (B) and inflammation score. For the associations between glucose measures and low-grade inflammation, solid arrows represent the total effect (ie, the association between 2h-PG or HbA1c and inflammation score), while dashed arrows indicate the direct effect (ie, the association between 2h-PG or HbA1c and inflammation score that is not attributable to MGO); a × b is the indirect effect via (changes in) plasma MGO. The proportion mediated effect (a × b/c) was calculated only when the total effect (e) and indirect effect (a × b) were significant.

Subsequent mediation analyses showed that 2h-MGO was a significant mediator in the associations of both 2h-PG and HbA1c with the low-grade inflammation score (Fig. 1). 2h-MGO mediated 23.5% of the association between 2h-PG and low-grade inflammation, and 16.4% of the association between HbA1c and low-grade inflammation. In the mediation analyses of association between the 2h-PG or HbA1c and the individual markers of low-grade inflammation (Tables S3 and S5 (20)), the mediating effect of 2h-MGO was the strongest in the association of 2h-PG with IL-8, where 2h-MGO mediated 78.5% of the association. The proportion mediated by 2h-MGO in the association between HbA1c and IL-8 was 31.3%, and for the association with CRP was 11%.

Sensitivity analyses in which participants without CVD and participants without diabetes were excluded, respectively, did not materially change the results of these mediation analyses (Figure S2 (20)).

## Discussion

In this large population-based cohort, we investigated the hypothesis that glucose excursions are more strongly associated with low-grade inflammation than fasting glucose and that these associations are mediated by postglucose load MGO concentrations. The main findings were that both 2h-PG and HbA1c, but not FPG, were independently associated with low-grade inflammation and that 2h-MGO partially mediated these associations.

2h-PG and HbA1c were independently associated with low-grade inflammation, with a somewhat stronger association for 2h-PG. This is line with previous data that an oral glucose challenge acutely induces the expression of proinflammatory genes in leukocytes of healthy individuals (25). Moreover, in a 14-year follow-up evaluation of persons with T2DM, HbA1c and 2h-PG but not FPG predicted CVD events and all-cause mortality (26), while 2h-PG but not FPG or HbA1c predicted future cardiovascular events in patients with coronary artery disease (27).

For FPG, our observed associations with inflammation were not consistent across persons with and without T2DM. While FPG was inversely associated with low-grade inflammation in persons without T2DM, this association was positive in those with T2DM. Interestingly, it has been shown that also in persons with normal glucose tolerance, both continuously elevated glucose levels for 5 hours and repeated glucose peaks during this time frame induce plasma inflammatory cytokines, an observation that was more pronounced in those persons with impaired glucose tolerance (10). In this experiment the height of the increase in glucose levels of continuous elevation and of the induced peaks were similar. It is therefore possible that the continuously higher FPG concentrations in our dataset were high enough to induce an inflammatory response in persons with T2DM. Alternatively, it is possible that the elevated FPG in persons with T2DM partly reflects previous glucose excursion that have not returned to normal levels.

Interestingly, MGO was associated with inflammation independent of plasma glucose levels. We previously showed that, even in normoglycemic mice, MGO injections induce elevations of monocytes and neutrophils, vascular inflammatory gene expression, and increased atherosclerotic burden (28). Moreover, we found that higher plasma MGO levels are associated with incident CVD and mortality (29), and in patients with severe limb ischemia with mortality, amputations, and with more inflammation and circulating white blood cells (30). Mechanistically, several studies have shown that MGO induces inflammation as MGO promotes monocyte chemotactic protein-1 expression in endothelial cells through activation of p38 (31) and induces expression of IL-1β, TNF, and IL-8 in a human macrophage cell line (32). In addition, the fast generation of AGEs by MGO (17) may add to the inflammatory effect of MGO, potentially via interaction with RAGE. In endothelial cells, RAGE activates NF-κB leading to transcription of inflammatory mediators such as IL-1β, IL-6, and TNF (33, 34). Furthermore, MGO induced cell death in macrophages, and accumulation of MGO-derived AGEs in human atherosclerotic plaques is associated with plaque inflammation (35).

Given the close relationship between postprandial glycemic excursions and accumulation of plasma MGO (15, 16, 36) and given that plasma MGO is derived from exogenous glucose during an OGTT in humans (17), our data support a pathway where inflammation caused by high blood sugar levels can occur via the production of MGO. This pathway could potentially contribute to an elevated risk of CVD.

Interestingly, the mediating effect of MGO in the association of glucose excursions and postload glucose with low-grade inflammation was mainly driven by IL-8. The associations of 2h-PG and HbA1c with IL-8 were comparable to the other cytokines, while, in contrast, the glucose-independent association of 2h-MGO with IL-8 was stronger than for the other cytokines. IL-8 is involved in neutrophil activation and it has been shown that both IL-8 and neutrophils were increased upon an acute glucose challenge (37), although this association is not consistently observed for postprandial glucose and neutrophil activation (38-40). Thus, the exact mechanisms behind the strong mediating effect of MGO on the relationship between peak glucose and IL-8 as well as the potential involvement of neutrophil activation in hyperglycemia-induced inflammation remain to be investigated.

This study has several strengths. The Maastricht Study is a large population-based cohort study with extensive phenotyping, allowing thorough adjustment for a substantial number of potential confounders. In addition, we measured plasma glucose and MGO both fasting and after a glucose challenge. Furthermore, MGO levels were measured using state of the art methods. This study also has limitations. First, although we adjusted for many confounders, we cannot rule out the possibility of residual confounding. In addition, inflammation markers were measured only in fasting plasma. It would be preferable if these markers were available at both fasting and 2-hour postload time points for corresponding analyses. Moreover, we estimated glucose excursions based on 2h-PG levels during an OGTT, while continuous glucose monitoring would provide more accurate data on glucose variability. Since 2h-PG is considered to approximate peak values in people with diabetes and therefore is a reasonable assessment of postprandial hyperglycemia (41), we used this time point to measure MGO levels and perform mediation analyses. In addition, we used the 2008 WHO definition of prediabetes and diabetes. There are other definitions of diabetes status based on FPG or HbA1c (42). We cannot exclude that the fact that we found interaction of our analysis only for T2DM, but not prediabetes, may depend on the clinical definition of prediabetes. Lastly, the participants were primarily of European descent, aged between 40 and 75 years, and extrapolating the current results to other ethnicities and ages should, therefore, be done with caution.

In conclusion, in a large-scale population-based cohort study, we found that peak rather than fasting glucose levels were associated with inflammation, and that MGO mediated this relationship. These data indicate that the postprandial MGO formation from glucose, contributes to the low-grade inflammation that can predispose to cardiovascular risk. Lowering postprandial MGO formation may therefore be a promising strategy to reduce chronic inflammation and cardiovascular diseases.

## Data Availability

Some or all datasets generated during and/or analyzed during the current study are not publicly available but are available from the corresponding author on reasonable request.

## Abbreviations

2h-PG: 2-hour plasma glucose
AGE: advanced glycation endproduct
BMI: body mass index
CRP: C-reactive protein
CVD: cardiovascular disease
DHD: Dutch Healthy Diet
FPG: fasting plasma glucose
HbA1c: glycosylated hemoglobin A1c
IL: interleukin
MGO: methylglyoxal
NGM: normal glucose metabolism
OGTT: oral glucose tolerance test
RAGE: receptor for AGE
T2DM: type 2 diabetes mellitus
TNF: tumor necrosis factor
